# Identification and Characterization of pantocin wh-1, a Novel Cyclic Polypeptide Produced by *Pantoea dispersa* W18

**DOI:** 10.3390/molecules25030485

**Published:** 2020-01-23

**Authors:** Tieshan Teng, Xianghui Li, Lei Zhang, Yanzhang Li

**Affiliations:** 1 Joint National Laboratory for Antibody Drug Engineering, Institute of Biomedical Informatics, School of Basic Medical Sciences, Henan University, Kaifeng 475004, China; xiaoshan1220@163.com (T.T.); xianghui_li0813@163.com (X.L.); zhlei@henu.edu.cn (L.Z.); 2 State Key Laboratory of Virology, Wuhan Institute of Virology, Chinese Academy of Sciences, Wuhan 430071, China

**Keywords:** Pantoea dispersa, peptide natural products, Mycobacterium tuberculosis

## Abstract

*Pantoea dispersa* W18, isolated from contaminated soil, was found to exert antimicrobial activity against Mycobacterium species, including *Mycobacterium tuberculosis*, an important human pathogen. Here, the anti-mycobacterial compound produced by *Pantoea dispersa* W18 was purified by a combination of hydrophobic interaction chromatography, cation exchange chromatography, and reverse phase HPLC. Active compounds from *Pantoea dispersa* W18 were identified as a natural peptide named pantocin wh-1 with a 1927 Da molecular weight. The primary structure of this compound was detected by N-terminal amino acid sequencing. The amino acid sequence of pantocin wh-1 consisted of 16 amino acid residues with a cyclic structure. The pantocin wh-1 could be inactivated by protease K, but was heat stable and unaffected by pH (2–12). However, the activity was not completely inactivated by trypsin and pepsin. This is the first report of a cyclic polypeptide purified from a strain of *Pantoea dispersa*.

## 1. Introduction

Microbially derived natural products have revealed enormous reservoirs of as yet untapped lead compounds. These natural products, possessing a wide variety of biological activities such as antibacterial and antiviral, endow them with remarkable applications in medicine. Ribosomally synthesized and post-translationally modified peptides (RiPPs), which have garnered considerable recent attention, are attributed to one class of microbially derived natural products [1]. The structural features of RiPPs consist of a precursor peptide including a C-terminal core region with a variety of modification sites and an N-terminal leader-peptide sequence [2]. Owing to the rise of antibiotic resistant infections, there is an urgent need for novel RiPPs, which hold promise for diseases with limited treatment options [3]. Several methods of arousing silent genes, including in situ cultivation, ichip, nutrient limitation, and environmental stresses, have been reported to obtain novel lead bio-active substances from microbial communities [4]. In this paper, we screen extracts from *Pantoea dispersa* against Mycobacteria and identify pantocin wh-1, a bio-active substance that was produced depending on nutrient limitation stress.

*Pantoea dispersa*, also known as members of the “*Erwinia herbicola-Enterobacter agglomerans* complex”, can be isolated from plant surfaces, seeds, soil, water, as well as animal and human wounds, blood, and urine [5]. Members of the *Pantoea* group are considered as good producers of natural products, such as organic acid [6], hydroquinone [7], and bacteriocins [8], which confer the *Pantoea* group a double advantage in terms of their survival in different habitats (Table 1). Although a diverse array of nature products generated by the genus *Pantoea* has been reported, most of them were extracted from *Pantoea agglomerans* as biological control agents for fire blight [9]. In addition, a few natural products were reported from *Pantoea dispersa* as antituberculosis agents. Here, a novel natural product named pantocin wh-1 was characterized, as well as evaluated for its anti-tuberculosis activity in vitro and in vivo.

## 2. Results

### 2.1. Isolation and Taxonomical Identification of the Natural Product Producing Strain

**Figure 1:**
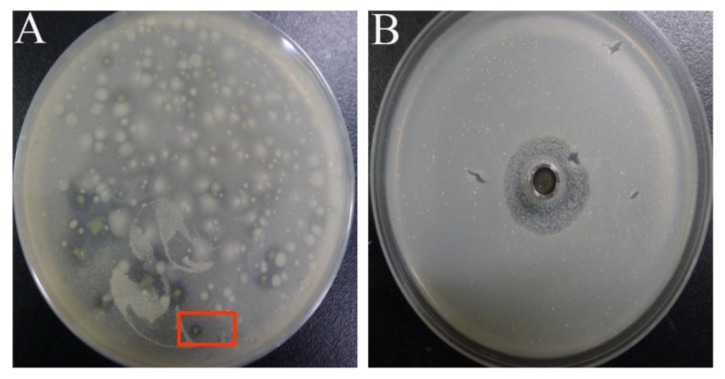
(**A**) On a plate of *M. smegmatis*, a yellow colony is shown to secrete active substances to inhibit the growth of *M. smegmatis* mc^2^ 155; (**B**) using the oxford cup method, the cell-free supernatant (CFS) of the fermentation broth of this unknown strain exhibits antibacterial activity against *M. smegmatis* mc^2^ 155.

On the plate of *M. smegmatis* mc^2^ 155, a significant inhibition zone formed by a yellow colony was observed by accident (red square, Figure 1A). This colony was picked for pure culture, and the cell-free supernatant (CFS) exhibited significant antimicrobial activity against *M. smegmatis* mc^2^ 155 and sensitivity in the face of proteinase K (Figure 1B). The 16SrDNA sequence of this strain revealed 100% similarity with *P. dispersa* by using BLAST alignment in NCBI. Furthermore, the Microbial Identification and Phenotype Microarray System results were in agreement with it being a microbial *P. dispersa* species, which verified that this strain belonged to *P. dispersa*.

### 2.2. The Production of Antimicrobial Peptide in Different Media and Physical Conditions

**Figure 2:**
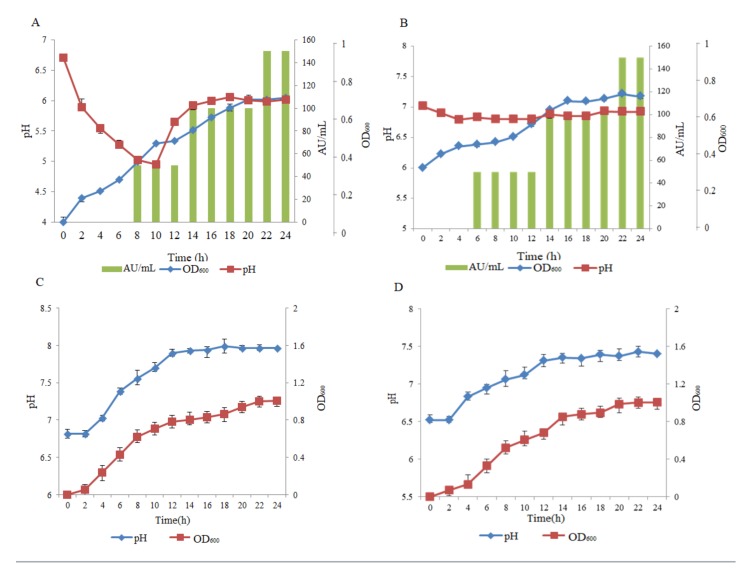
Time course of pantocin wh-1 production during the growth of *P. dispersa* W18 in: (**A**) simple 7H9 medium; (**B**) Spizizen’s medium; (**C**) LB medium; and (**D**) MH medium. The amount of pantocin wh-1 is expressed as arbitrary units per milliliter (AU/mL). The indicator strain was *M. smegmatis* mc^2^ 155.

All three media, including lysogeny broth (LB), 7H9, and Spizizen’s minimal medium (SMM), were used to evaluate the effect of the production of pantocin wh-1. The results showed that pantocin wh-1 could be secreted at the exponential phase of *P. dispersa* W18 in 7H9 and Spizizen’s minimal medium and reach a maximum value at the late stationary phase (Figure 2A,B). On the other hand, Both in LB medium and MH medium, the production of pantocin wh-1 was not observed, although *P. dispersa* W18 could grow better than in the other two mediums (Figure 2C and 2D). This result may indicate that pantocin wh-1 was secreted in oligotrophic habitats for biological competition, but not in nutrient rich environment habitats.

In the presence of 7H9 medium, the pH value of the CFS was gradually reduced from 7 to 4.8 along with *P. dispersa* W18’s growth and finally stabilized at about 6. In contrast, in LB medium, the pH value of the supernatant was gradually increased from 7 to 8. However, the pH value had almost no change in Spizizen’s minimal medium. When *P. dispersa* W18 was grown in 7H9 medium, the CFS had the optimal antibacterial activity against *M. smegmatis* mc^2^ 155. The optimal condition for pantocin wh-1 production was *P. dispersa* W18 cultured in 7H9 medium at 30 °C for 48 h with shaking at 166 rpm.

### 2.3. Purification of pantocin wh-1 and Estimation of the Molecular Weight

The initial crude extract of antimicrobial peptide was obtained by ultrafiltration, phenol-chloroform extraction, and vacuum lyophilization. Subsequently, the three methods consisting of cation exchange chromatography, hydrophobic interaction, and reverse phase HPLC were used to purify the polypeptide produced by *P. dispersa* W18. In the final reverse phase HPLC step, anti-mycobacterial activity was observed by the sample collected at a retention time of 13.234 min (Figure S1). All the other eluted fractions at retention times of 2.363, 8.802, 10.351, 10.692, and 14.237 min were also collected respectively and exhibited no antibacterial activity against *M. smegmatis* mc^2^ 155. The pantocin wh-1 was characterized by mass spectrometry, and MALDI-TOF showed a molecular ion of 1927 Da (Figure S2C) and, after purification, a yield of 23% and a concentration of 0.3 mg/mL, as shown in Table 2. The MALDI-TOF mass spectrum of the crude extract from *P. dispersa* W18 is shown in Figure S2A. The major ion observed in MALDI-TOF had a molecular mass of 1927 Da. When treated with proteinase K, the antibacterial activity of pantocin wh-1 was inactivated completely, combined with the peak of 1927 Da *m*/*z* disappearing (Figure S2B). Disappointingly, when pantocin wh-1 was subjected to automated N-terminal amino acid sequencing by Edman degradation, no PTH-amino acid sequence was obtained, indicating that the N-terminus of pantocin wh-1 was blocked.

### 2.4. Fragmentation of pantocin wh-1

N-terminal Edman sequencing of purified pantocin wh-1 could not obtain an effective amino acid sequence, indicating that pantocin wh-1 could contain some amino acid modification or cyclization structure. For detecting the partial amino acid sequence of pantocin wh-1, various methods for fragmenting pantocin wh-1 including trypsin, BNPS-skatole, and hydrochloric acid were carried out (Table 3). Subsequently, the obtained fragments were purified by HPLC and subjected to amino acid sequencing. Two fragment peptides obtained after treatment with trypsin for 1 h were characterized by tandem mass spectrometry sequencing, providing the following primary sequences MWCYVF for 848.04 Da and WVMWCYVFG for 1190.43 Da. When treated with 0.6 M HCl, one fragment of peptides was obtained as follows: YVFGYGF for 851.94 Da. The pantocin wh-1 also was treated with BNPS-skatole, which cleaves polypeptides specifically at tryptophan residues. After that, one fragment with a molecular mass of 1163.3 Da was yielded, corresponding to the primary sequence WVMWCYVFG. Additionally, the sequences acquired from above were merged with each other, and the intact amino acid residues of pantocin wh-1 were chemically determined, consisting of 16 amino acid residues, and formed a circular polypeptide structure.

### 2.5. Effect of Various Enzymes, Heat, and pH on the Antibacterial Activity of pantocin wh-1

As shown in Table S1, the effects of various enzymes, heat treatment, and pH on the bactericidal activity of pantocin wh-1 were detected. The antibacterial activity of pantocin wh-1 was insensitive to lysozyme, DNase I, and RNase A. The inhibitory activity of pantocin wh-1 was inactivated completely by proteinase K and showed partial sensitivity to trypsin. pantocin wh-1 was heat stable after incubation at 100 °C for 30 min and retained 72% activity by autoclaving at 121 °C for 30 min. Furthermore, the antibacterial activity of pantocin wh-1 also retained nearly 100% of the initial activity within a broad pH range (pH 2–12). The above results exhibited that pantocin wh-1 showed a high degree of stability against pH, heat treatment, and high resistance to proteases (except for proteinase K), which was in line with other cyclic polypeptides.

### 2.6. Antibacterial Spectrum and Time Kill Curve

**Figure 3:**
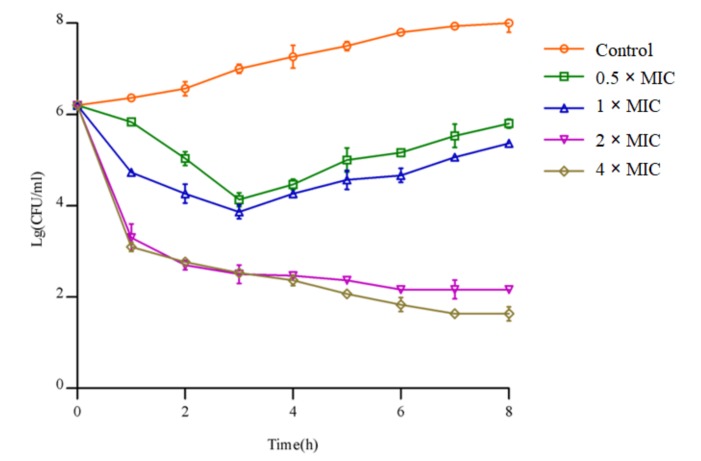
Bactericidal activity of pantocin wh-1 against *M. smegmatis* mc^2^ 155.

In this paper, the microporous dilution method was used to detect the antibacterial spectrum of pantocin wh-1 against Gram-negative and -positive bacteria. As shown in Table S2, in addition to partial inhibitory activity against *Klebsiella*, *Listeria monocytogenes*, and *Streptococcus suis*, pantocin wh-1 exhibited persistent antibacterial activity against mycobacterial specificity. In vitro, the MIC values of pantocin wh-1 against *M. smegmatis* mc^2^ 155 and *M. tuberculosis* H37Ra were 2.5 μg/mL and 7.5 μg/mL, respectively. The time kill studies were determined over 8 h with *M. smegmatis* mc^2^ 155 being exposed to 0.5, 1, 2, or 4 × MIC of pantocin wh-1. At 0.5 and 1 × MIC, a rapid and significant decline (>3 log drop) in colony forming units (CFU) within the first 2 h was observed, following a bacterial regeneration to near the initial concentration (Figure 3). At 2 × MIC and 4 × MIC, pantocin wh-1 caused bacterial eradication was completed approximately after 4 h.

**Figure 4:**
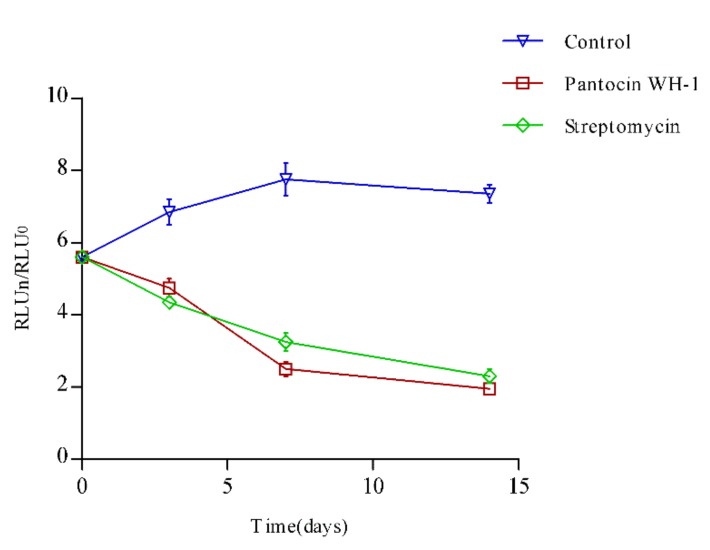
Antimicrobial activity of pantocin wh-1 in a mouse model.

The anti-mycobacterial activity of pantocin wh-1 was further assessed in vivo using BALB/c female mice infected with autoluminescent H37Ra (UAlRa). The anti-tuberculosis drug streptomycin was used as a positive control, and carboxymethylcellulose sodium (NaCMC) was used as a negative control. All pantocin wh-1, streptomycin, and NaCMC were formulated into 30 μg/mL, and each 200 μL were injected into the experimental and control mice each day for six consecutive days. The relative light unit (RLU) was detected every two days. As shown in Figure 4, on the second day of treatment, the RLU value exhibited a significant decrease. However, the therapeutic effect of pantocin wh-1 did not exceed that of streptomycin in the final day. The peptide drugs could be hydrolyzed by proteases in vivo, resulting in shorter half-lives. Hence, the therapeutic effect of pantocin wh-1 in a mouse model may be further improved by increasing the half-life of pantocinwh-1.

## 3. Discussion

The increasing clinical resistance of *M. tuberculosis* to conventional anti-tuberculosis drugs resulted in a growing interest in considering natural products as alternative anti-tuberculosis agents. The silent metabolic pathway of microorganisms can be activated by various culture methods to produce a large number of natural products with extensive biological activity, which is an important source of lead compounds. For TB treatment, microorganism derived antimicrobial peptides have been reported widely, such as teixobactin, ecumicin, lassomycin, etc. (Table 4). These natural products with the characteristics of peptides possess unique targets for killing *M. tuberculosis* without affecting other microorganisms.

Changes in the composition of the medium can affect the type and relative yield of the metabolites of the strain. In this study, *P. dispersa* W18 could produce pantocin wh-1 in modified 7H9 and SMM medium, but not in LB medium. We hypothesized that the lower nutrient condition could mimic the growth environment of *P. dispersa* W18 in nature and activate silent metabolic pathways to synthesize novel products. pantocin wh-1 showed inhibitory activity against Mycobacteria in vitro and in vivo. The result of the antibacterial spectrum test exhibited that the antibacterial activity against Mycobacteria was specific, indicating that an unusual target for action may be present in *M. tuberculosis* and needs to be explored in future trials like screening mutant libraries.

Cysteine residues were presented in the amino acid sequence of pantocin wh-1 and were easy to oxidize to form a disulfide bond, giving rise to dimers or oligomers. Therefore, NMR (nuclear magnetic resonance) techniques need to be performed to verify the formation of disulfide bonds in the next step. In previous experiments, pantocin wh-1 was detected to have two antibacterial mechanisms, ROS (reactive oxygen species) production and cell wall destruction, which were dependent on the pH value. However, whether two antibacterial mechanisms are related to the formation of the disulfide bond remains to be verified.

## 4. Materials and Methods

### 4.1. Bacterial Strains and Culture Conditions

*M. smegmatis* mc^2^ 155, M. bovis BCG, and *M. tuberculosis* H_37_Ra were grown in Middlebrook’s 7H9 broth medium supplemented with 10% OADC (oleic acid–albumin–dextrose–catalase) and 0.05% Tween 80 at 37 °C without shaking. *M. smegmatis* mc^2^ 155 was used as the indicator strain for the inhibitory activity assay. *P. dispersa* W18 was grown in a modified Middlebrook’s 7H9 broth medium and 10% OADC substituted with 1% d-glucose. The *Escherichia coli* strain DH5α was grown in lysogeny broth (LB) medium, and positive transformants were selected on LB agar plates containing 30 mg/liter ampicillin.

### 4.2. Identification of the pantocin wh-1 Producing Strain

Two methods were performed to identify the pantocin wh-1 producing strain: 16S rDNA sequencing analysis and the Microbial Identification and Phenotype Microarray System (MIPM, GENIII Omnilog II Combo plus System, BIOLOG, Winooski, VT, USA) [26]. Two 16S rDNA universal primers 27F/1492R (5′-AGAGTTTGATCCTGGCTCAG-3′; 5′-TACGGCTACCTTGTTACGACTT-3′) were synthesized for PCR identification. The PCR cycling protocol consisted of pre-denaturation at 94 °C for 5 min, 30 cycles of denaturation at 94 °C for 30 s, annealing at 55 °C for 45 s, extension at 72 °C for 1.5 min, and repair at 72 °C for 10 min. For the microarray method, a single *P. dispersa* W18 colony was picked with a cotton swab, gently rubbing the IF-1A tube wall, and the colony was evenly dispersed in the IF-1A solution. Then, the solution was inoculated into a 96 well GenIII plate, incubated at 37 °C for 24 h, and the value was read by a microplate reader.

### 4.3. Purification of pantocin wh-1

To obtain a cell-free supernatant (CFS) of *P. dispersa* W18, this strain was cultivated in 7H9 medium at 37 °C for 18 h. The cells were removed by centrifugation at 8000× *g* for 10 min at 4 °C, filtered with 0.22 μm pore size filters, and extracted with phenol-chloroform (1:1). Then, the upper aqueous phase was performed by an ultrafiltration procedure through a variety of ultrafiltration units with 50 KDa, 30 KDa, 10 KDa, and 3 KDa molecular cut-offs (Millipore, billerica, MA, USA). The active fraction was obtained by stepwise ultrafiltration, lyophilized, and weighed. The obtained active retentate was loaded onto a Sepharose™ FF cation exchange column (Sigma) pre-equilibrated with Buffer A (50 mM sodium citrate, pH = 3). The bound fraction was subsequently eluted with 1 M sodium chloride in Buffer A. Furthermore, the active fraction was loaded onto an Octyl-Sepharose^®^ CL-4B column (Sigma) pre-equilibrated with Buffer B (1 M NH_4_SO_4_ in 50 mM sodium phosphate) [27]. Then, the active polypeptide eluted with 70% ethanol in Buffer B was lyophilized and weighed. The active lyophilized fraction was re-suspended in 10% acetonitrile containing 0.1% TFA, and the concentration was adjusted to 30 μg/mL. Then, the sample was subjected to the high performance liquid chromatography (HPLC) system with a C18 reverse phase column. Elution was performed with acetonitrile in water containing 0.1% TFA, using a linear gradient from 10 to 80% for 35 min, at a flow rate of 1 mL/min. The sample injection volume was 20 μL. The eluted samples were detected using a UV detector at 215 nm and 280 nm. Each peak was collected, and a rotary evaporator was used to remove the acetonitrile component, while the antibacterial activity was determined against *M. smegmatis* mc^2^ 155.

The purified pantocin wh-1 was lyophilized and stored at −20 °C. The anti-mycobacterial active fraction obtained in each step was detected as described above by using *M. smegmatis* mc^2^ 155 as an indicator strain. The protein concentration of each fraction was examined using a NanoDrop ND-1000 Spectrophotometer.

### 4.4. Mass Spectrometry Analysis

Mass spectrometric analysis of pantocin wh-1 was performed through an ABI4800 MALDI-TOF device with a 337 nm nitrogen laser for desorption/ionization. The saturated matrix solution used for MALDI-TOF MS was α-cyano-4-hydroxycinnamic acid (CHCA) in 50% aqueous acetonitrile containing 0.1% *v*/*v* TFA [28,29]. Positive ion detection and linear mode were used for MALDI-TOF mass spectra. Proteolytic digestion of pantocin wh-1 was monitored with a Bruker Autoflex Speed MALDI-TOF/TOF mass spectrometer. The sample preparation method was the same as that outlined above.

### 4.5. Fragmentation of pantocin wh-1

Trypsin, hydrochloric acid, and BNPS-skatole (2-(2-nitrophenylsulfenyl)-3-methyl-3′-bromoindolenine) were performed with pantocin wh-1 to obtain a part of the amino acid sequence. The purified pantocin wh-1 was dissolved in 200 μL 50% acetonitrile containing 0.1% TFA. For the proteolytic digestion of pantocin wh-1 with trypsin, a 50 μL aliquot of pantocin wh-1 was mixed with 50 μL of 50 mM NH_4_HCO_3_ (pH 7.5). Then, samples were digested by the addition of 10 μL trypsin solution consisting of 2 μg trypsin. Proteolytic digestion fragmentation of pantocin wh-1 was determined by MALDI-TOF MS at 0, 4, 8, 12, and 24 h at 37 °C. The presence of BNPS-skatole led to cleavage of the polypeptide with the C-terminal side of the tryptophan residue. Lyophilized pantocin wh-1 was dissolved in sodium citrate solution containing BNPS-skatole. The reaction was treated at 37 °C for 72 h, and the product was fractionated by HPLC as described above and used for subsequent analyses. Lyophilized pantocin wh-1 was also dissolved in 0.6 M HCl at a final concentration of 0.2 mg/mL and then incubated at 37 °C for 20 h.

### 4.6. Effect of Various Enzymes, Heat, and pH on the Bactericidal Activity of pantocin wh-1

The effect of various enzymes, pH, and temperature on pantocin wh-1 activity was detected as described by J.-M. Chobert et al. [30] with a slight modification. Proteinase K, trypsin, catalase, lysozyme, DNAse, and RNAse were used to test the antibacterial activity of pantocinwh-1. To analyze the effect of different pH values on the antibacterial activity of pantocin wh-1, the sample of pantocin wh-1 was exposed to buffer with a pH value of 2–12 for 1 h at 25 °C. The effect of temperature on the anti-mycobacterial activity of pantocin wh-1 was determined by incubating pantocin wh-1 at 50 °C and 100 °C for 60 min, as well as at 121 °C for 15 min, and then detecting the bactericidal activity against *M. smegmatis* mc^2^ 155.

### 4.7. Detection of Antibacterial Activity

The antibacterial activity of the pantocin wh-1 was detected by the microscale optical density assay (MODA) method [31,32]. Briefly, 90 μL of the dilutions of pantocin wh-1, 100 μL 2 × 7H9 medium, and 10 μL of a log phase indicator strain were co-incubated in the 96-well plate at 37 °C for 48 h. For control groups, 90 μL of the dilutions of pantocin wh-1 were replaced by an equal volume of sterile water. After incubation, the 96 well microplate was measured by a microplate reader at a 600 nm wavelength (Biotek synergyH1, Biotek, Winooski, VT, USA). The amount of pantocin wh-1 produced by *P. dispersa* W18 was determined using two fold dilutions. One arbitrary unit (AU) was defined as the reciprocal of the highest dilution that resulted in the inhibition of the growth of *M. smegmatis* mc^2^ 155. The results were expressed in AU/mL. The MIC was defined as the last dilution of pantocin wh-1 sufficient to prevent indicator strain growth in vitro.

For detection of the time kill study, 100 μL of the indicator strain in exponential phase were added to tubes containing 5 mL of the modified 7H9 medium with pantocin wh-1 at 0.5, 1, 2, and 4 × MIC. No pantocin wh-1 was added in the control tube, then incubated at 37 °C for 8 h. At the end of each time period, tenfold serial dilutions were prepared with 0.5 mM phosphate buffer, and 100 μL cultures were plated in triplicate onto the 7H10 plates. For anti-tuberculosis activities in vivo, an auto-luminescent strain of *M. tuberculosis* H37Ra (AlRa) described by Tianyu Zhang [33] was used to assess the efficacy of pantocin wh-1 in a mouse model. The relative light unit (RLU) count was determined to parallel CFU counts during the active phase of bacterial growth. AlRa with OD_600_ of 0.1 was injected into female BALB/c mice by tail vein. The day after injection (Day 0), RLU values were detected for 6 days in triplicate [34].

## Figures and Tables

**Table 1:** Active natural products secreted by *Pantoea* species.

Strain	Name	Structure	Ref.
*P. agglomerans* EH318	pantocin B	(*R*)-*N*-[((*S*)-2-amino-propanoylamino)-methyl]-2-methanesulfonyl-succinamic acid	[10]
	pantocin A	unknown	
*P. agglomerans* Eh335	andrimid	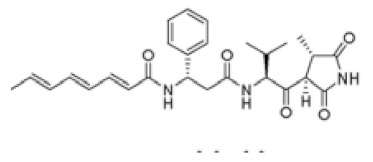	[11]
*P. agglomerans* C9-1/48b/90		2-amino-3-(oxirane-2, 3-dicarboxamido)-propanoyl-valine	[12]
*P. agglomerans* C9-1	herbicolin O	unknown	
	herbicolin I	unknown	
*P. agglomerans* Eh1087	phenazine	d-alanylgriseoluteic acid (AGA)	[13]
*E. herbicola* 112Y	112Y	unknown	
*E. herbicola* A 111	herbicolin A	(*R*)-3-OH-Cl_4_-DH-Abu-l-Thr-d-aThr-d-Leu-Gly-d-Glx-Gly-l-Me-aThr-L-Arg	[14]
	herbicolin B	d-Abu-l-Thr-d-aThr-d-Leu-Gly-d-Gln-Gly-*N*-Me-l-aThr-l-Arg	

**Table 2:** Purification of pantocinwh-1 produced by *Pantoea dispersa* W18.

Method	Vol(ml)	Total Activity (AU) ^1^	Yield (%)	Total Protein (mg) ^2^	Purification (fold)
Supernatant	2000	1.60 × 10^6^	100	16,520	1
SP-Sepharose	350	5 × 10^5^	80	3537	75
Octyl-Sepharose	120	5 × 10^5^	80	623	212
RP-HPLC	4	2.3 × 10^5^	23	1.52	963

^1^ Antibacterial activity (expressed in arbitrary units (AU)) was assayed by the spot-on-lawn method using *M. smegmatis* mc^2^ 155 as an indicator strain. ^2^ The protein concentration (in mg/mL) was estimated with a NanoDrop ND-1000 (A280).

**Table 3:** Amino acid sequences of pantocin wh-1 fragments obtained after different fragmentation strategies.

Treatment	Molecular Mass of Fragment (Da)	Amino Acid Sequence
0.6 M HCl	851.94	YVFGYGF
BNPS-skatole	1163.3	FGYGFNCAVW
Trypsin	848.04	MWCYVF
	1190.43	WVMWCYVFG
Merge	1927.76	—VMWCYVFGYGFNCAVW—

**Table 4:** Source, activity, and target of anti-TB natural products.

Compound	Source	Strategy	Target	MIC (μM)	Ref.
Lassomycin	*Lentzea kentuckyensis* sp.	In situ cultivation and prolonged incubation	ClpC1	0.42–1.57	[15,16]
Ecumicin	*Nonomuraea* sp., strain MJM5123	High-throughput screening and stress of nutrient limitation	ClpC1	0.13–0.3	[17]
Acyldepsipeptides	*Streptococcus hawaiiensis*	Stress of nutrient limitation	ClpP	31–65	[18,19]
Pyridomycin	*Dactylosporangium fulvum*	Genetic manipulation	InhA	0.72–1.44	[20]
Thiolactomycin	*Nocardia* sp.	Genetic manipulation	β-ketoacyl-ACP synthase	92.5	[21]
Cyclomarin A	*Streptomyces* sp.	Stress of nutrient limitation	ClpC1	0.3–2.5	[22,23]
Teixobactin	*Eleftheria terrae*	ichip	lipid II	0.125	[24]
Ilamycins	*Streptomyces* sp. SCSIO16	Gene inactivation and isotope labeled	—	0.0098	[25]

## Supplementary Materials

The following are available online, Figure S1: (a) Further purification of pantocin wh-1 was performed by HPLC with a C18 reverse column, and the fraction at 13.234 min had anti-mycobacterial activity; (b) a linear gradient used in this study, Figure S2: (a) The upper figure shows that the molecular weight of the pantocin wh-1 sample was 1927 Da using MALDI-TOF/MS analysis; (b) the middle figure shows that the molecular weight of pantocin wh-1 treated with proteinase K was changed to 1456 Da; (c) the lower figure shows the molecular weight of pantocin wh-1 purified by HPLC, Table S1: Effects of enzymes, heat, and pH on pantocin wh-1 activity, Table S2: Antimicrobial spectrum of pantocin wh-1 produced by *P. dispersa* W18.
