# Supplementary material for: Identification and Characterization of pantocin wh-1, a Novel Cyclic Polypeptide Produced by Pantoea dispersa W18
Source: Molecules. 2020 Jan 23;25(3):485. doi: 10.3390/molecules25030485 (PMC7036871; doi:10.3390/molecules25030485)
Supplement: Supplementary file 1 [file molecules-25-00485-s001.pdf]

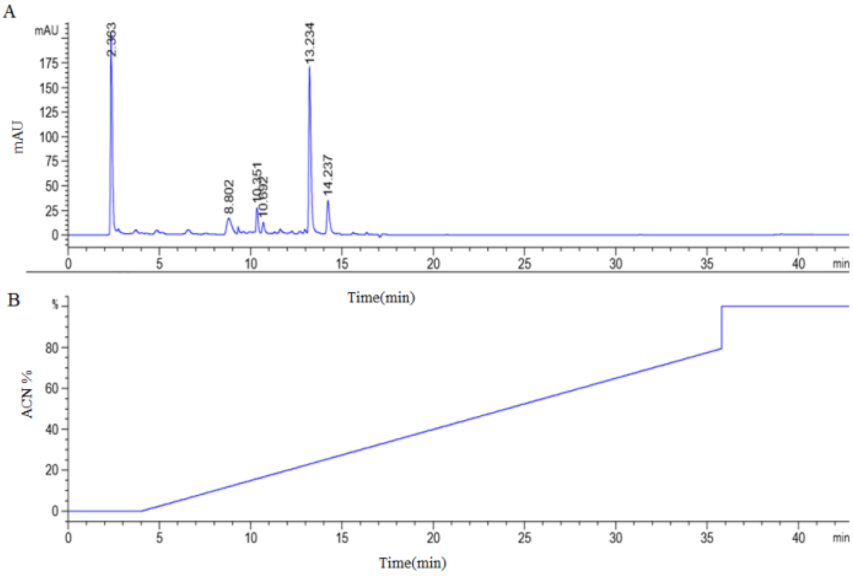

Figure S**1.** **(a)** Further purification of PantocinWH-1 was performed by HPLC with a C18 reverse column and the fraction at 13.234min has anti-mycobacterial activity; **(b)** a linear gradient used in this study.

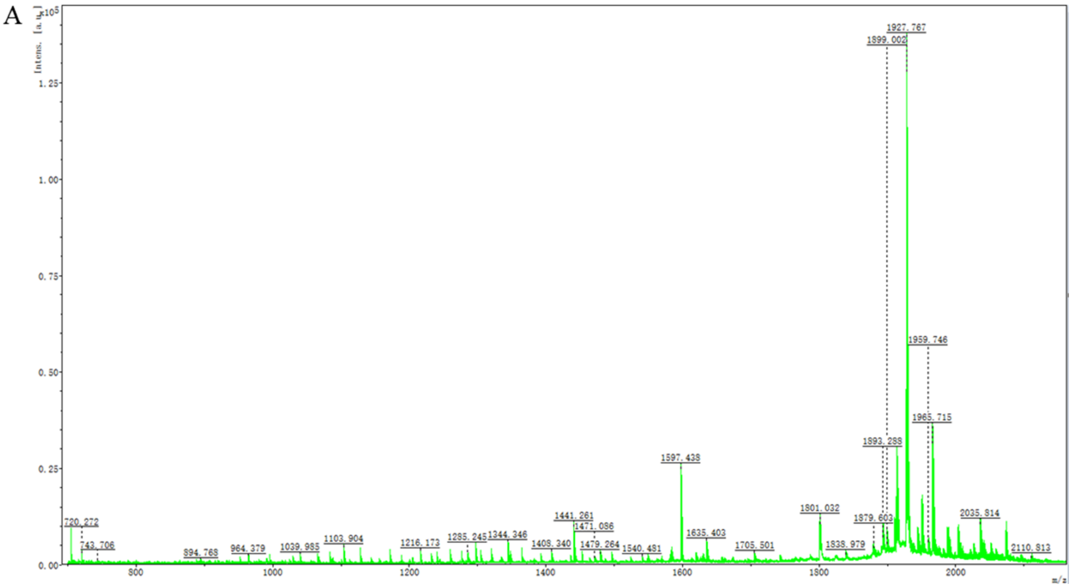

B

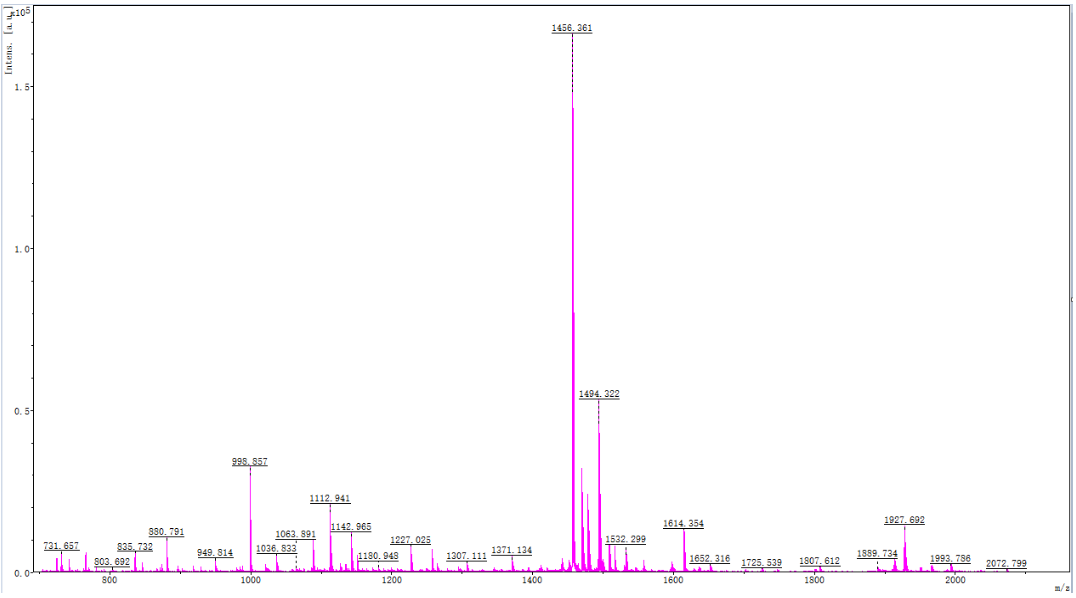

C

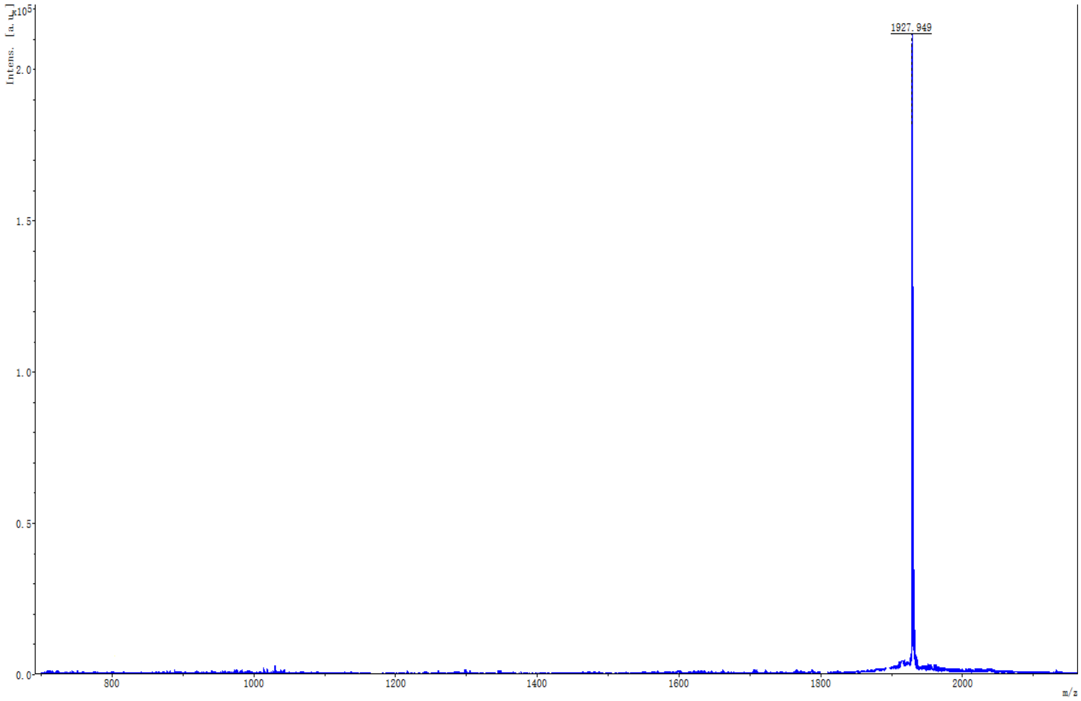

Figure S2. **(a)**The upper figure showed that the molecular weight of PantocinWH-1sample was 1927Da using MALDI-TOF/MS analysis; **(b)** the middle figure showed that the molecular weight of PantocinWH-1 treated with proteinase K was changed to 1456Da; **(c)** the lower figure showed that the molecular weight of PantocinWH-1 purified by HPLC .

Table S1. Effects of enzymes, heat and pH on PantocinWH-1 activity

	Treatment	Residual activity (%)[1]
Enzymes	proteinase K	20
	lysozyme	100
	trypsin	27
	DNase	100
	RNase	100
Heat	50℃/ 30 min	100

	100℃/ 30 min	90
	121℃/ 15 min	72
pH	pH2-6	90
	pH6-8	100
	pH8-12	85

[1] Residual activity compared with antimicrobial activity before the treatment

Table S**2.** Antimicrobial spectrum of PantocinWH-1 produced by *P. dispersa* W18

**Indicator organism**	**Medium**	**Inhibition activity**[1]
Bacillus cereus	LB	-
S. aureus B31	LB	-
S. aureus B30	LB	-
S. aureus AM025	LB	-
S. aureus N315	LB	-
S. aureus 21A	LB	-
White aureus 8799	LB	-
Feces Enterococcus	BHI	-
L .monocytogenes	BHI	+
S. suis	BHI	+
Dysgalactiae	BHI	-
S. pyogenes	BHI	-
M. smegmatis mc^{2}155	7H9	+++
M. tuberculosis Ra	7H9	+++
M. bovis BCG	7H9	+++
E. coli O157	LB	-
P. aeruginosa	LB	-

[1] -, no inhibitory activity; +, Inhibit the growth of less than 10% amount of the initial bacteria; ++, Inhibit the growth of less than 50% amount of the initial bacteria; +++, Inhibit the growth of initial bacteria completely.
